# Supplementary material for: Measurement properties of the 30-second sit-to-stand test in post COVID-19 condition: Results from the PYCNOVID randomised controlled trial
Source: PLoS One. 2026 May 12;21(5):e0348275. doi: 10.1371/journal.pone.0348275 (PMC13166962; doi:10.1371/journal.pone.0348275)
Supplement: S1 Table — (DOCX) [file pone.0348275.s001.docx]

**Supplementary Table**

**Table S1. Participant characteristics at baseline.**

| **Characteristics** | **All (n=150)** |
| --- | --- |
| **Education level** |  |
| Primary | 4 (2.7) |
| Upper secondary | 42 (28.0) |
| Tertiary | 104 (69.3) |
| **Smoking status** |  |
| Never | 96 (64.0) |
| Former | 42 (28.0) |
| Current | 12 (8.0) |
| **Patient reported outcomes** | |
| FACIT-Fatigue score <34 | 124 (82.7) |
| MoCA score <26 | 19 (12.7) |
| HADS – Anxiety score ≥7 | 61 (40.7) |
| HADS – Depression score ≥7 | 68 (45.3) |
| Post-exertional malaise | 141 (94.0) |
| Mild | 14 (9.9) |
| Moderate | 58 (41.1) |
| Severe | 54 (38.3) |
| Very severe | 15 (10.6) |

Data are presented as number (percentages). HADS, Hospital, Anxiety and Depression Scale; MoCA, Montreal Cognitive Assessment Test. Post-exertional malaise was assessed on a 5-point Likert Scale with anchors ranging from “not bad at all” to “very severe”.
