# Supplementary material for: Measurement properties of the 30-second sit-to-stand test in post COVID-19 condition: Results from the PYCNOVID randomised controlled trial
Source: PLoS One. 2026 May 12;21(5):e0348275. doi: 10.1371/journal.pone.0348275 (PMC13166962; doi:10.1371/journal.pone.0348275)
Supplement: S2 Table — (DOCX) [file pone.0348275.s002.docx]

**Supplementary Table**

| **Characteristics** | **Maximal effort (n=116)** | **Submaximal effort (n=34)** |
| --- | --- | --- |
| Female sex, n (%) | 85 (73.3) | 27 (79.4) |
| Age, years | 45.1 ± 12.9 | 42.8 ± 1.4 |
| Hospitalized for SARS-CoV-2 infection, n (%) | 5 (4.3) | 1 (2.9) |
| Time since onset of symptoms, weeks | 114 ± 62.5 | 101 ± 38.3 |
| **Comorbidities** |  |  |
| Cardiovascular Disease, n (%) | 4 (3.4) | 3 (8.8) |
| Hypertension, n (%) | 9 (7.8) | 2 (5.9) |
| Diabetes, n (%) | 3 (2.6) | 0 (0) |
| Obesity, n (%) | 21 (18.1) | 7 (20.6) |
| Chronic Respiratory Disease, n (%) | 18 (15.5) | 3 (8.8) |
| Chronic Kidney Disease, n (%) | 2 (1.7) | 1 (2.9) |
| Autoimmune Disease, n (%) | 5 (4.3) | 2 (5.9) |
| Psychiatric Disease, n (%) | 19 (16.4) | 1 (2.9) |
| **Patient-reported outcomes** |  |  |
| FACIT-Fatigue | 23.5 ± 9.5 | 24.1 ± 8.2 |
| MoCA | 27.5 ± 1.9 | 27.9 ± 1.6 |
| CRQ – Fatigue | 3.5 ± 1.0 | 3.6 ± 1.0 |
| CRQ – Dyspnoea | 5.7 ± 1.2 | 5.7 ± 1.2 |
| CRQ – Emotional | 4.5 ± 0.9 | 4.8 ± 1.0 |
| CRQ – Mastery | 5.7 ± 0.9 | 5.7 ± 1.0 |
| HADS – Anxiety | 6.16 ± 3.96 | 5.26 ± 3.15 |
| HADS – Depression | 6.62 ± 3.77 | 6.35 ± 3.91 |
| EQ-5D-5L index | 0.59 ± 0.24 | 0.62 ± 0.18 |
| EQ-VAS | 51.2 ± 20.2 | 48.0 ± 16.6 |
| **Physical activity (n = 139)** |  |  |
| ENMO, mg | 9.31 ± 3.62 | 9.22 ± 3.72 |
| Inactivity, min.day^-1^ | 1050 ± 101 | 1040 ± 92 |
| Light PA, min.day^-1^ | 26 ± 10 | 29 ± 8 |
| Moderate PA, min.day^-1^ | 48 ± 26 | 48 ± 28 |
| Vigorous PA, min.day^-1^ | 0.9 ± 2.0 | 0.4 ± 1.0 |

**Table S2.** Descriptive characteristics of the groups with maximal and submaximal effort in the 30-second sit-to-stand test (30s-STS).

Data are presented as number (percentages) or mean ± standard deviation (SD). CRQ, Chronic Respiratory Questionnaire; ENMO, Eucledian Norm Minus One; EQ-5D-5L; EuroQol 5-Dimension 5-Level, EQ-VAS; EuroQol Visual Analogue Scale (0-100); HADS, Hospital, Anxiety and Depression Scale; MoCA, Montreal Cognitive Assessment Test; PA, physical activity.
