# Supplementary material for: Measurement properties of the 30-second sit-to-stand test in post COVID-19 condition: Results from the PYCNOVID randomised controlled trial
Source: PLoS One. 2026 May 12;21(5):e0348275. doi: 10.1371/journal.pone.0348275 (PMC13166962; doi:10.1371/journal.pone.0348275)
Supplement: S3 Table — (DOCX) [file pone.0348275.s003.docx]

**Supplementary Table**

**Table S3.** 30-second sit-to-stand data at 12 weeks and change from baseline to 12 weeks.

|  | **12 weeks**  **(n=144)** | **Change**  **(n=144)** |
| --- | --- | --- |
| Repetitions, n | 16.6 ± 6.6 | 0.8 ± 3.0 |
| Repetitions, z-score | -1.3 ± 1.2 | 0.1 ± 0.5 |
| Heart rate – pre-test, beats.min^-1^ | 74 ± 10 | -0.4 ± 10.2 |
| Heart rate – post-test, beats.min^-1^ | 103 ± 17 | 3.2 ± 14.5 |
| SpO_2_ – pre-test, % | 98 ± 1 | 0.1 ± 1.5 |
| SpO_2_ – post-test, % | 98 ± 2 | -0.2 ± 1.6 |
| Dyspnoea – pre-test, 0-10 scale | 0.7 ± 1.0 | 0.04 ± 0.94 |
| Dyspnoea – post-test, 0-10 scale | 2.6 ± 1.7 | -0.1 ± 1.5 |
| Leg fatigue – pre-test, 0-10 scale | 1.5 ± 1.6 | -0.3 ± 1.6 |
| Leg fatigue – post-test, 0-10 scale | 3.4 ± 2.0 | -0.3 ± 1.7 |

Data are presented as mean ± standard deviation. SpO_2_, oxygen saturation. Dyspnoea and leg fatigue were assessed on a 0-10 Borg scale.
