# Supplementary material for: Measurement properties of the 30-second sit-to-stand test in post COVID-19 condition: Results from the PYCNOVID randomised controlled trial
Source: PLoS One. 2026 May 12;21(5):e0348275. doi: 10.1371/journal.pone.0348275 (PMC13166962; doi:10.1371/journal.pone.0348275)
Supplement: S4 Table — (DOCX) [file pone.0348275.s004.docx]

**Supplementary Table**

**Table S4.**  Minimal important difference (MID) values used for the potential anchors and associated number of changers (n=144).

| **Instrument** | **MID** | **Changers (worse)** | **Changers (better)** |
| --- | --- | --- | --- |
| CRQ Dyspnoea* | 0.5 | 25 | 35 |
| FACIT Fatigue | 2.7 | 40 | 71 |
| HADS total | 1.5 | 40 | 64 |
| HADS anxiety | 1.5 | 30 | 48 |
| HADS depression | 1.5 | 22 | 49 |
| EQ-VAS* | 8 | 27 | 60 |

CRQ, Chronic Respiratory Questionnaire; EQ-VAS, EuroQol Visual Analogue Scale; HADS, Hospital Anxiety and Depression Scale; MID, minimal important difference. *Data available for 142 participants.
