# Supplementary material for: Measurement properties of the 30-second sit-to-stand test in post COVID-19 condition: Results from the PYCNOVID randomised controlled trial
Source: PLoS One. 2026 May 12;21(5):e0348275. doi: 10.1371/journal.pone.0348275 (PMC13166962; doi:10.1371/journal.pone.0348275)

**Supplementary Figure**

**Figure S1.** Bland-Altman plot for the agreement between 30-second sit-to-stand (30s-STS) repetitions at screening and at baseline, excluding participants with submaximal effort. The blue line represents the mean bias of 1.17 (95% confidence interval from 0.48 to 1.87). The dashed lines represent the lower and upper limit of agreement, with respective values of -6.26 (95% confidence interval from -7.46 to -5.07) and 8.61 (95% confidence interval from 7.41 to 9.80).


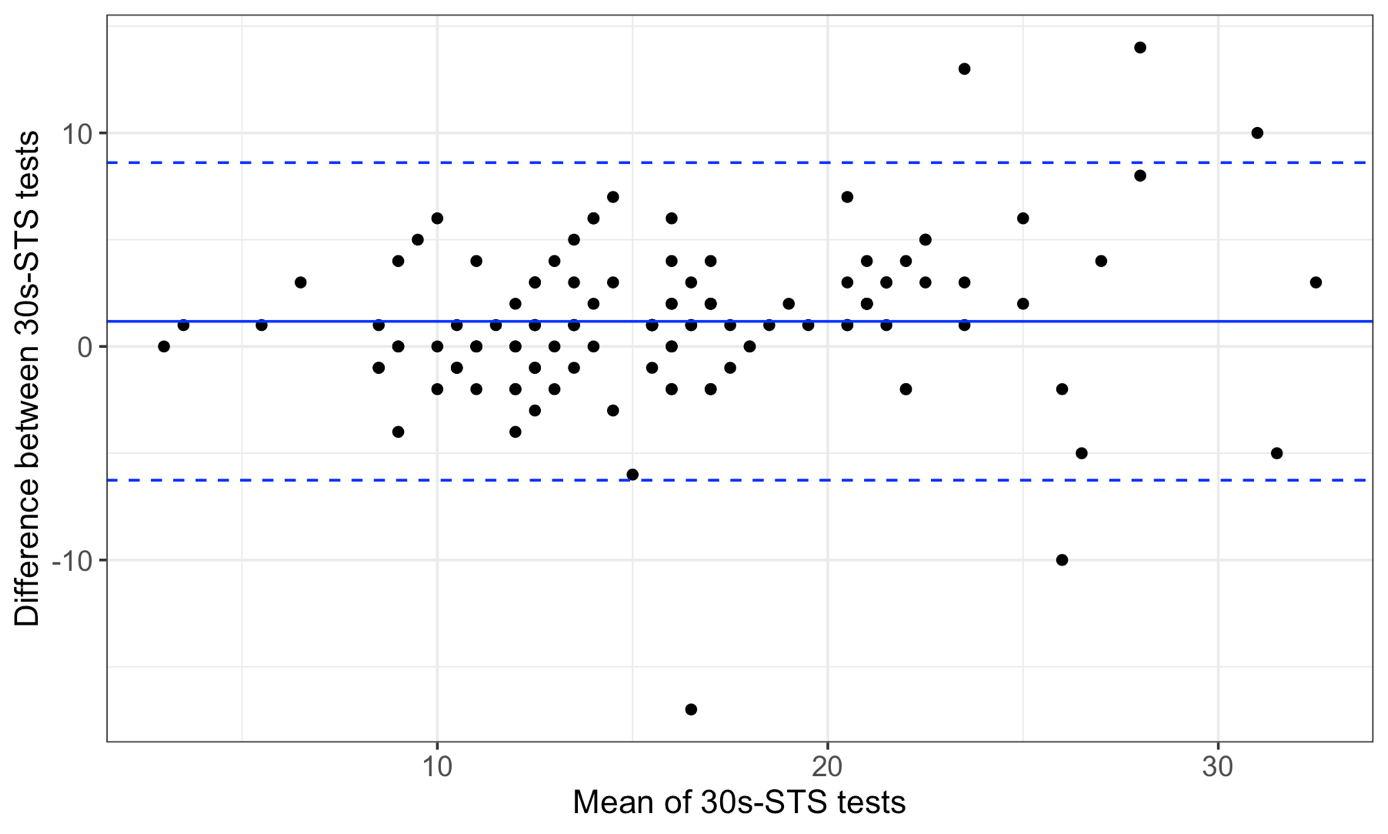

Supplement: S1 Fig — The blue line represents the mean bias of 1.17 (95% confidence interval from 0.48 to 1.87). The dashed lines represent the lower and upper limit of agreement, with respective values of −6.26 (95% confidence interval from −7.46 to −5.07) and 8.61 (95% confidence interval from 7.41 to 9.80). (DOCX) [file pone.0348275.s005.docx]
