# Supplementary material for: Measurement properties of the 30-second sit-to-stand test in post COVID-19 condition: Results from the PYCNOVID randomised controlled trial
Source: PLoS One. 2026 May 12;21(5):e0348275. doi: 10.1371/journal.pone.0348275 (PMC13166962; doi:10.1371/journal.pone.0348275)
Supplement: S2 Fig — Spearman correlation coefficients were as follows: r = 0.26 (A), r = 0.22 (B), r = 0.20 (C), and r = 0.22 (D). (DOCX) [file pone.0348275.s006.docx]

**Supplementary Figure**

**Figure S2.** Scatterplots showing relationships at baseline between 30-second sit-to-stand test (30s-STS) z-scores and daily steps (A), Eucledian Norm Minus One (ENMO) values (B), time spent in daily moderate physical activity (C), and time spent in daily vigorous physical activity (D). Spearman correlation coefficients were as follows: r=0.26 (A), r=0.22 (B), r=0.20 (C), and r= 0.22 (D).

**
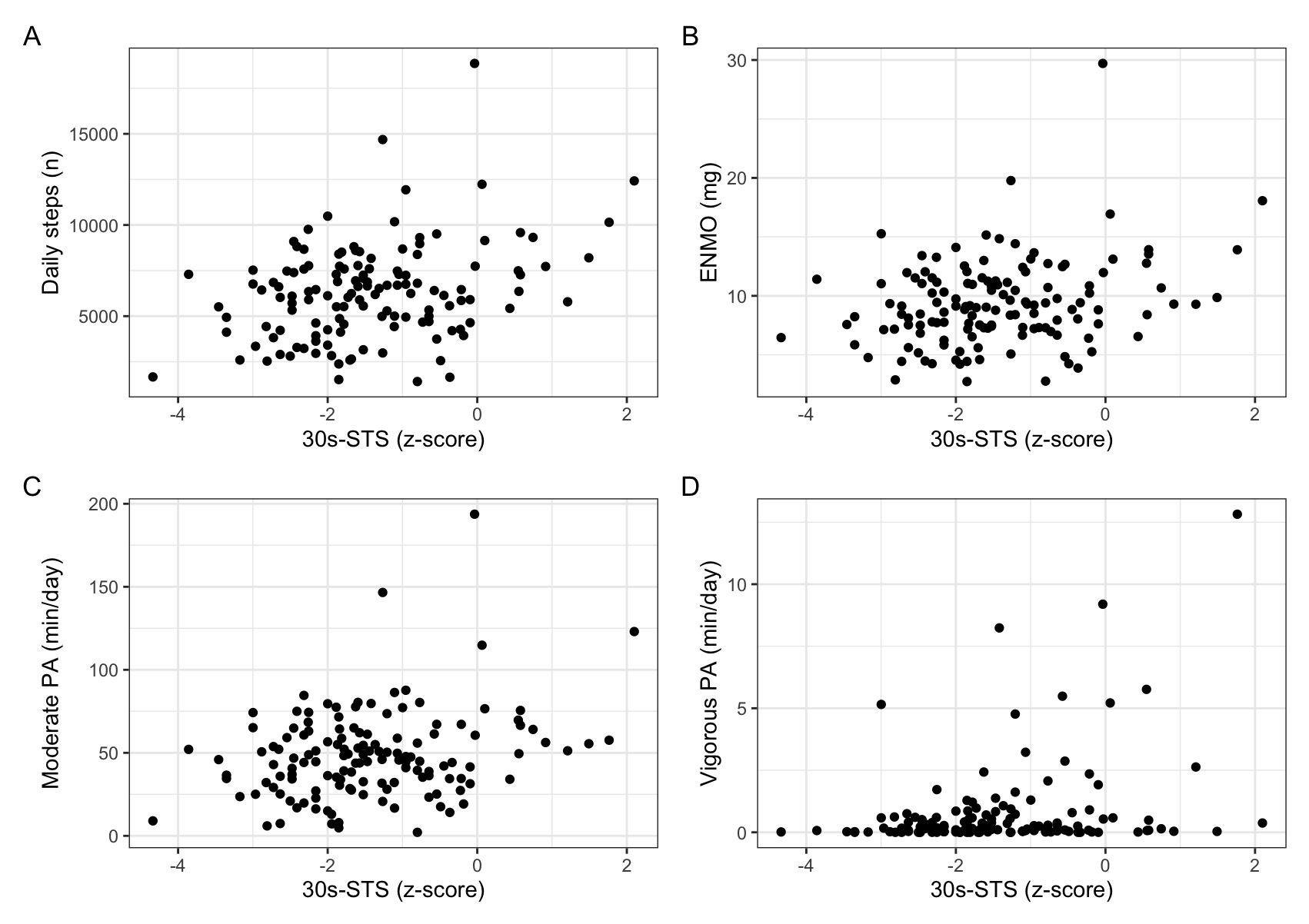
**
