# Supplementary material for: Measurement properties of the 30-second sit-to-stand test in post COVID-19 condition: Results from the PYCNOVID randomised controlled trial
Source: PLoS One. 2026 May 12;21(5):e0348275. doi: 10.1371/journal.pone.0348275 (PMC13166962; doi:10.1371/journal.pone.0348275)

**Supplementary Figure**

**Figure S3. A)** Boxplots and **B)** Q-Q plots for the assessment of the distribution of change from baseline to 12 weeks of the anchors and 30s-STS repetitions.


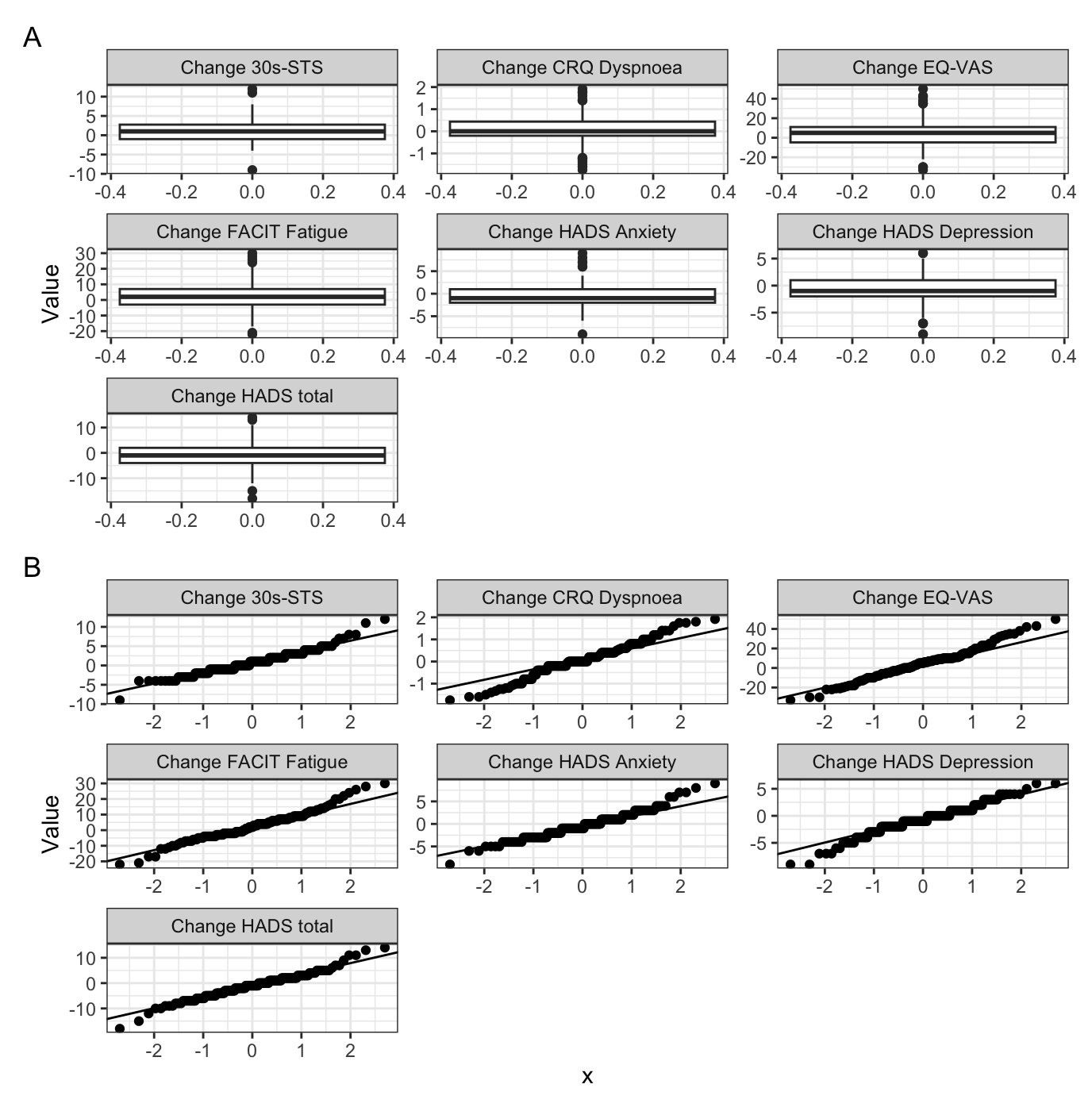

Supplement: S3 Fig — (DOCX) [file pone.0348275.s007.docx]
