# Supplementary material for: Measurement properties of the 30-second sit-to-stand test in post COVID-19 condition: Results from the PYCNOVID randomised controlled trial
Source: PLoS One. 2026 May 12;21(5):e0348275. doi: 10.1371/journal.pone.0348275 (PMC13166962; doi:10.1371/journal.pone.0348275)
Supplement: S4 Fig — (DOCX) [file pone.0348275.s008.docx]

**Supplementary Figure**

**Figure S4.** Scatterplots of the relationship between the change of the anchor variables and the 30s-STS repetitions between baseline and 12 weeks.

**
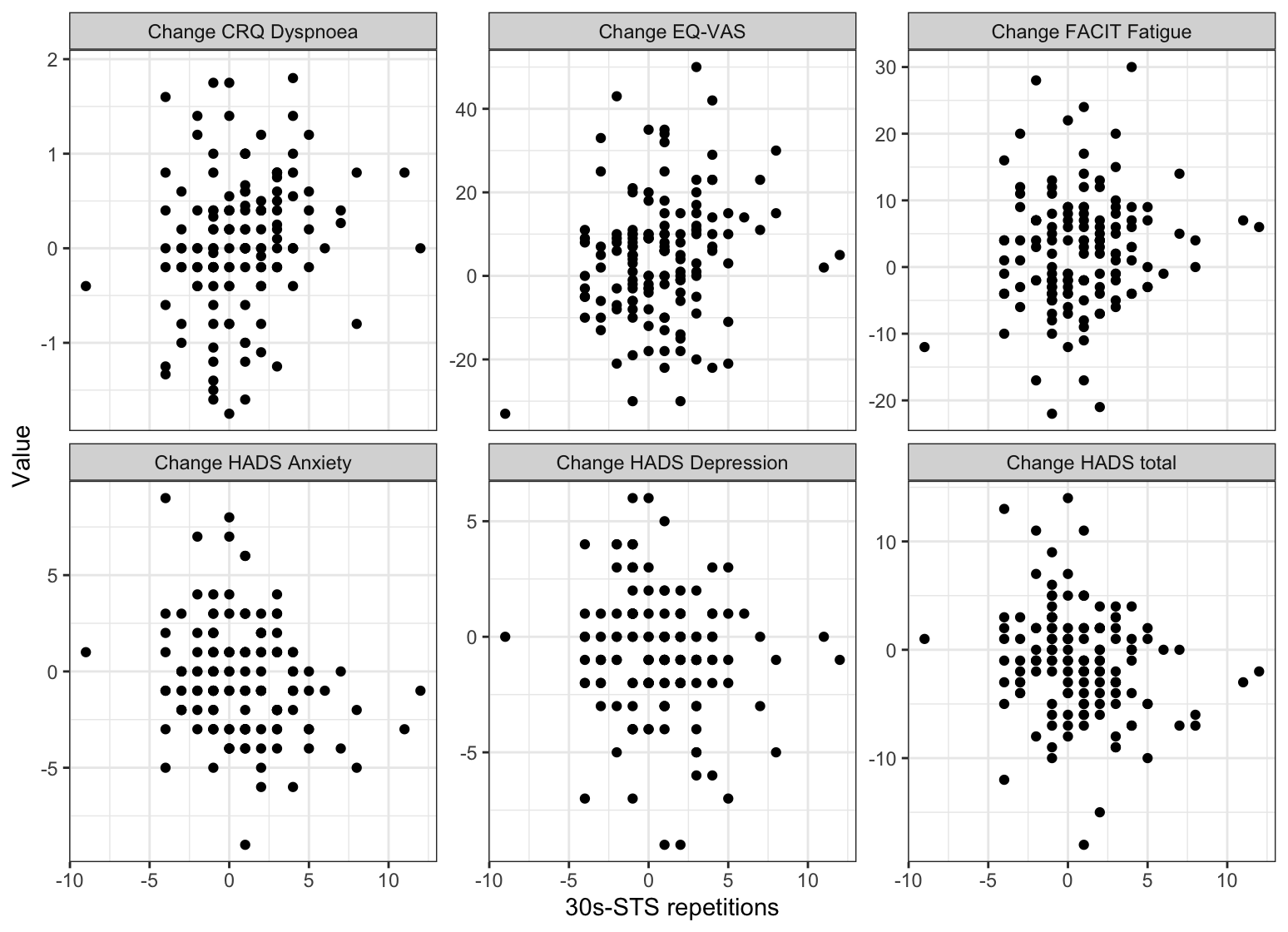
**
