# Supplementary material for: Measurement properties of the 30-second sit-to-stand test in post COVID-19 condition: Results from the PYCNOVID randomised controlled trial
Source: PLoS One. 2026 May 12;21(5):e0348275. doi: 10.1371/journal.pone.0348275 (PMC13166962; doi:10.1371/journal.pone.0348275)
Supplement: Attachment — Submitted filename: PONE-D-26-04488_Response to Reviewers_20260330.pdf [file pone.0348275.s012.pdf]

PONE-D-26-04488

We thank the reviewers and editors for their careful review of our manuscript. The manuscript has undergone substantial revision, including additional statistical analyses, incorporating all reviewer comments. We provide point-by-point responses to each of the reviewers' questions and hope that we have addressed all points to their satisfaction.

Review Comments to the Author

Reviewer #1: This manuscript examines the 30-second sit-to-stand (30s-STS) test in individuals with post-COVID-19 condition (PCC). Overall, the study is well conducted, clearly reported, and methodologically sound.

However,
1) The manuscript reports weak correlations between 30s-STS performance and key PCC-related symptoms, stating that this was "expected." However, the theoretical basis for this expectation is not sufficiently articulated. The authors should more clearly define and explain why weak correlations are theoretically plausible.

Response: Thank you for this comment. PCC is a multisystem condition with more than 200 reported symptoms and multiple phenotypes. In some patients, symptoms such as fatigue, post-exertional malaise, or dyspnoea are likely to be associated with performance in the sit-to-stand test, whereas in others this may not be the case due to substantial heterogeneity in symptom profiles and severity across patients. Consequently, when assessed across a heterogeneous population, these variable and patient-specific associations are likely to result in overall weak correlations.

We added the following paragraph to the methods section (pages 11-12):

2.4.2 Construct validity

"Validity is a measure of how well a test measures what it sets out to measure, that is, if it relates to the gold standard measure (criterion validity) or other measures that assess the same construct.

Regarding construct validity, potential relationships between z-scores of the 30s-STS test and PEM, fatigue, dyspnoea, and physical activity were investigated using boxplots and scatterplots, along with the calculation of Spearman's correlation coefficients. We formulated a priori hypotheses regarding the expected direction and strength of correlation. PCC is a multisystem condition with more than 200 reported symptoms and multiple phenotypes [34,35]. In some patients, symptoms such as fatigue, post-exertional malaise, or dyspnoea are likely to be associated with performance in the 30s-STS test, whereas in others this may not be the case due to substantial heterogeneity in symptom profiles and severity. Consequently, when assessed across a heterogeneous population, these variable and patient-specific associations are likely to result in overall weak correlations.

We anticipated that higher symptom burden and lower physical activity would be associated with lower 30s-STS performance. However, given that the 30s-STS tests

reflects integrative functional capacity, we expected these correlations to be weak to at most moderate, as symptoms, and physical activity are not direct measures of functional capacity and lower limb muscle function. Furthermore, we hypothesised that subgroups of people with severe-to-very severe PEM, which represents a significant barrier to sustained (structured) physical activity, would demonstrate the lowest 30s-STS performance.

As closely related objective measures of lower-limb muscle function were not available, convergent validity could only be assessed indirectly. Therefore, the examined variables were expected *to show weak correlations, reflecting related but distinct constructs".*

2) Considering 22.7% of participants reported submaximal effort due to fear of symptom exacerbation. This is a clinically important issue in PCC. While a sensitivity analysis is reported, the authors should explicitly discuss how this behaviour affects the validity and clinical interpretation of the 30s-STS.

Response: We thank the reviewer for this comment and have extended the DISCUSSION section to cover validity aspects and clinical interpretation of individual test results. The new paragraph reads (page 19-20):

"Importantly, almost one quarter reported limiting their effort out of fear that symptoms might exacerbate, influencing their 30s-STS results. This group had remarkably lower 30s-STS z-scores compared to those who reported maximum effort (-2.11 versus -1.23). We did not identify any characteristics that explain these findings, such as more severe symptom burden, lower physical activity levels, or presence of chronic disease(s) other than PCC. The two groups were well comparable with respect to demographic and clinical characteristics and had reported similar quality of life (Table S2). Notably, this aspect is important to consider because some people with PCC may be capable of performing better in the 30-s STS but chose not to, due to fear of symptom exacerbation ("crashes"). Consequently, submaximal effort may lead to an underestimation of individual functional performance, which has implications for the validity of the test results at the individual level and their clinical interpretation. From a clinical perspective, it is therefore important to assess fear of symptom exacerbation as part of exercise testing in people with PCC".

3) The images in the figures are blurry; please upload sharp versions of these images.

We are sorry that the figures were of insufficient quality. We provide figures with higher resolution and hope that they now are more clearly visible.

Reviewer #2: Please describe the measurement properties you evaluated in the methodology section. And explain in detail how you evaluated them. You haven't provided any information about these. Also, which methodological criteria did you use? For example, the most commonly used guideline currently is the COSMIN checklist. Did you base your analysis on this? For instance, COSMIN does not recommend analyzing psychometric properties using data from previously conducted longitudinal studies. For detailed information, please refer to the guideline at cosmin.nl.

Response: We thank the reviewer for their careful review and for providing critical but constructive feedback. While the COSMIN guidelines were originally developed for patient-reported outcome measures, many of their domains are applicable to performance-based functional tests. For the 30s-STS, we applied relevant COSMIN domains, including reliability, measurement error, construct validity, and responsiveness, while acknowledging that some items, particularly those assessing content validity, are less applicable to a single-task functional assessment. This approach allowed us to evaluate the measurement properties rigorously while remaining consistent with widely accepted methodological standards.

Please find attached the COSMIN reporting guideline (Version 2.0, Gagnier et al., Quality of Life Research, 2025), indicating where each item is addressed in the manuscript. Please note that COSMIN no longer recommends use of the original checklist (Mokkink et al., 2010). We therefore used the recently published reporting guideline from Gangnier and colleagues.

Page 14: "The methodology for assessing reliability, validity, reliability, and responsiveness of the 30s-STS test were guided by COSMIN recommendations, with adaptations made to account for the fact that the 30s-STS is a functional capacity test rather than a patient-reported outcome measure".

We would like to emphasize that this work is a secondary analysis of data from a large placebo-controlled randomised trial. Although the original trial was not specifically designed to assess the measurement properties, the study design – including a screening and baseline visit about 2 weeks apart and the follow-up visit at 12 weeks – enabled us to study the feasibility, safety, reliability, measurement error, construct validity and responsiveness of the 30s-STS test in individuals with PCC.

Given the limited data on the measurement properties of the 30s STS test, we believe that our study provides important evidence for researchers and healthcare professionals caring for people with PCC. We acknowledge the limitations of our study, such as the provisional estimate of the distribution-based MID and limitations in construct validity, and we provide clear suggestions for future studies designed specifically to evaluate measurement properties, including appropriate endpoints for anchor-based analysis.

At what time intervals did you examine the test-retest reliability? Before and 12 weeks later? If so, that's a very significant time gap and not appropriate.

Response: Test-retest reliability was examined using STS data from screening and baseline visits (about 2 weeks apart). The 12-week assessment was used for the

calculation of a minimal important difference, but not for test-retest reliability analyses. The information is available in different sections of the revised manuscript.

You mentioned in the abstract that you looked at construct validity, but the methodology doesn't specify what you compared it to. What hypotheses do you have for divergent validity or convergent validity? Example article: Tolk et al. Measurement properties of the OARSI core set of performance-based measures for hip osteoarthritis: a prospective cohort study on reliability, construct validity and responsiveness in 90 hip osteoarthritis patients.

Response: We have added information on construct validity to the abstract, i.e., comparison to key PCC outcomes and physical activity. We extended the METHODS section on construct validity and acknowledge limitations. It reads (pages 11-12):

2.4.2 Construct validity

"Validity is a measure of how well a test measures what it sets out to measure, that is, if it relates to the reference standard measure (criterion validity) or other measures that assess the same construct.

"Regarding construct validity, potential relationships between z-scores of the 30s-STS test and PEM, fatigue, dyspnoea, and physical activity were investigated using boxplots and scatterplots, along with the calculation of Spearman's correlation coefficients. We formulated a priori hypotheses regarding the expected direction and strength of correlations. PCC is a multisystem condition with more than 200 reported symptoms and multiple phenotypes [34,35]. In some patients, symptoms such as fatigue, post-exertional malaise, or dyspnoea are likely to be associated with performance in the 30s-STS test, whereas in others this may not be the case due to substantial heterogeneity in symptom profiles and severity. Consequently, when assessed across a heterogeneous population, these variable and patient-specific associations are likely to result in overall weak correlations.

We anticipated that higher symptom burden and lower physical activity would be associated with lower 30s-STS performance. However, given that the 30s-STS tests reflects integrative functional capacity, we expected these correlations to be weak to at most moderate, as symptoms, and physical activity are not direct measures of functional capacity or lower limb muscle function. Furthermore, we hypothesised that subgroups of people with severe-to-very severe PEM, which represents a significant barrier to sustained (structured) physical activity, would demonstrate the lowest 30s-STS performance.

As closely related objective measures of lower-limb muscle function were not available, convergent validity could only be assessed indirectly. Therefore, the examined variables were expected to show weak correlations, reflecting related but distinct constructs".

Line 150: "...to assess a potential learning effect"... You are looking at reliability, and please remove this statement. Clearly specify the analysis sections for reliability or other measurement characteristics.

Response: We removed the term *learning effect*, as suggested. We extended the methods section on test-retest reliability. It reads:

2.4.1 Test-retest reliability

"Reliability refers to the consistency of a measurement and its ability to reproduce performance across repeated assessments. To assess test-retest reliability, we compared the 30s-STS results between screening and baseline using Bland-Altman plots and calculated one-way intraclass correlation coefficients (ICC) for consistency. We opted for the one-way ICC instead of the two-way ICC because the latter assumes that all participants' measurements were supervised by the same outcome assessors, which was not the case in our study.

Measurement error comprises systematic and random deviations in a participant's performance that are not attributable to true changes in the underlying construct. We calculated the standard error of measurement (SEM), defined as the standard deviation at screening times the square root of 1 minus ICC, and the minimal detectable change (MDC95), defined as 1.96 times the square root of 2 times SEM. The calculation of these two values gives additional information about the measurement error and its influence on change. As the screening 30s-STS test served as an initial test trial, this analysis also allowed us to examine whether a familiarisation test is necessary.

In a sensitivity analysis, we excluded participants with submaximal effort to evaluate the influence on test-retest reliability indices and assess the robustness of the primary analysis".

I don't mean to be disrespectful, but I think you're not generally familiar with this type of study design. You haven't explained the measurement characteristics, the analysis isn't appropriate, and you haven't followed a methodology. Nowadays, these kinds of validity and reliability studies undergo a rigorous methodology, and there are many examples of this in the literature.

Response: Team members from our group have been core investigators of large-scale international consortia to develop and validate PROs including the PROactive and Mobilise-D projects funded by the European Innovative Medicines Initiative. For example, PROactive, followed regulatory authority guidelines, integrated patient input, systematic literature reviews, created a conceptual framework, and used early validation studies for item reduction, confirmation of constructs, and assessment of measurement properties ultimately resulting in EMA approved clinical endpoints.

Further, we did thorough validation studies for the 1-min STS in people with chronic respiratory diseases and published in highly respected journals (e.g. Crook et al. Eur Respir J 2017) and have been involved in several validation studies of PROs over the last 20 years.

We honestly stated that this is a parent study of a randomised, placebo-controlled trial, and that the study was not designed specifically to assess measurement properties of the 30s-STS test.

The reviewer states that "the analysis isn't appropriate" but unfortunately provides no further clarification.

Test-retest reliability: We present ICCs (95% CIs) to evaluate relative reliability, and Bland-Altman analysis (mean bias and limits of agreement) to quantify absolute agreement. This combined approach is widely recommended for reliability studies on clinical and functional performance measures. The recommended article by Tolk et al. 2018 used similar statistical methods for test-retest reliability which is also supported by COSMIN guidance.

The revised manuscript includes information on measurement error (SEM) and the minimal detectable change at the 95 % confidence level (MDC95). Consistent with COSMIN guidance, the MDC95 was interpreted as the threshold beyond measurement error, while distribution-based methods are considered supportive.

Responsiveness to change: Responsiveness was evaluated using anchor- and distribution-based methods following established guidance (see references). Our approach aligns with COSMIN guidance, which prioritizes anchor-based methods for estimating the MID and considers distribution-based methods as supportive. As anchor correlations were insufficient in our study, MID estimated based on distribution-based studies are presented as provisional.

Reviewer #3: Dear Authors,

First, I would like to commend you for conducting this important and timely study. The manuscript presents a clearly structured investigation evaluating the measurement properties of the 30-second sit-to-stand (30s-STS) test in individuals with post-COVID-19 condition (PCC). The topic is clinically relevant and methodologically meaningful, particularly given the need for valid and responsive outcome measures in PCC rehabilitation.

While the study provides valuable insights, several areas require further clarification and scientific strengthening to meet the standards of a Q1-ranked journal. My detailed comments are outlined below.

Response. We are very grateful to the Reviewer for their exceptionally thorough and constructive feedback. The critical yet highly constructive comments have been invaluable, and we have carefully incorporated all suggestions, which we believe have substantially improved the manuscript.

Abstract

The background is appropriately introduced; however, the study aim should be stated more explicitly and concisely.

Response: We extended the study aims, as suggested. It reads: *"The aim of this study was to evaluate the measurement properties of the 30s-STS test for assessing*

functional capacity in adults with PCC. Specifically, we assessed it's the feasibility, test-retest reliability, construct validity, and responsiveness to change of the 30s-STS in a heterogeneous sample of individuals with PCC."

Lines 25–30: Please provide more detailed information about the study sample (e.g., recruitment source, key inclusion criteria, baseline severity).

Response: Thank you for this comment. We extended the paragraph and included details about inclusion/exclusion criteria and recruitment sources. It reads (pages 5-6):

«Individuals with PCC experiencing at least one persistent PCC symptom, such as fatigue, cognitive impairment ("brain fog"), dyspnoea, or PEM [20] were eligible to participate if they were aged 18 years or older, fluent in German, had no planned changes in medication, and had a confirmed SARS-CoV-2 infection verified by polymerase chain reaction (PCR), a rapid antigen test, or a physician's diagnosis of PCC. We applied the World Health Organisation definition of PCC [21] and assessed each participant's clinical history, including the timing of SARS-CoV-2 infection(s), vaccination(s), and the onset and progression of symptoms over time. Specifically, participants were required to have persistent symptoms for ≥12 weeks after SARS-CoV-2 infection that could not be explained by an alternative diagnosis. In the absence of a diagnostic biomarker, each case was carefully adjudicated based on medical report. Exclusion criteria comprised severe comorbidities such as renal failure or advanced heart failure, acute infections, untreated or unstable psychiatric disorders, recent COVID-19 vaccination (<4 weeks prior to baseline or during the study), intolerance to or regular use of the investigational product Pycnogenol®, or participation in another intervention study.

We recruited participants through various channels including the Altea network, the Long COVID Citizen Science Board, the Facebook group of Long COVID Schweiz, and advertisements in public transport in the city of Zurich and the surrounding metropolitan area. Furthermore, we shared flyers with different stakeholders, including clinics that care for individuals with PCC.

All participants provided written informed consent prior to enrolment".

You report "Mean bias 0.84 repetitions" and ICC (p1, l33–35). Consider including a clinically interpretable reliability metric (e.g., SEM or MDC95), as ICC alone is dependent on between-subject variability and may overestimate practical reliability.

Response: Thank you for this suggestion. We had now added both the SEM and the MDC95 values to the methods, results and discussion (see also our responses later):

Page 11: "Additionally, we calculated the standard error of measurement (SEM), defined as the standard deviation at screening times the square root of 1 minus ICC, and the minimal detectable change (MDC95), defined as 1.96 times the square root of 2 times SEM. The calculation of these two values gives additional information about the measurement error and its influence on change."

Page 19: "The relatively wide limits of agreement are in line with an SEM value of 2.35, suggesting that the true value of a 30s-STS measurement can lie within plus/minus 2.35 repetitions, and the associated MDC95 of 4.25.

Page 20: "However, measurement error and between-subject variability can influence test results which and should be considered when interpreting 30s-STS results."

Page 28: "Importantly, as the MDC95 of 4.25 repetitions suggests that changes below this threshold might be primarily or partly due to measurement error and between-subject variability, the proposed MID value of two repetitions must be interpreted with caution. A change of two repetitions could fall within measurement error, while changes above 4 repetitions can be interpreted as both real and meaningful. However, using a comparatively small MID of two repetitions for sample size calculations represents a conservative approach in the absence of further evidence: a smaller MID results in a larger sample size, thereby ensuring sufficient power for future studies."

Since anchor-based methods were unsuccessful, the term "minimal important difference" should be used more cautiously. It would be more precise to describe this as a "distribution-based estimate of minimal detectable/important change," unless a clear distinction between MID and MDC is consistently maintained throughout the manuscript.

Response: Thanks for this important comment. We ensure a clear distinction between the two terms throughout the manuscript, report data for minimal detectable change and the provisional distribution-based MID estimate.

Introduction

The rationale for assessing functional capacity in rehabilitation and core outcome sets is well presented. However, the current gap regarding 30s-STS measurement properties in PCC is not sufficiently developed. For a Q1-level manuscript, three short paragraphs are insufficient to fully justify novelty and contribution. The Introduction should more convincingly articulate why this investigation meaningfully advances the field.

Response: Thank you. We added the following paragraph to the INTRODUCTION section (page 4): It reads: *"Detailed knowledge of the measurement properties of the 30s-STS is critical for the interpretation of functional capacity (screening) and for evaluating changes in functional capacity following an intervention (responsiveness). Moreover, knowledge of estimates of meaningful change is a prerequisite for future trial design and sample size calculations."*

Specific suggestions:

Line 67: The transition to the STS test is appropriate; however, I strongly recommend briefly describing the biomechanical and physiological characteristics of the STS movement. Even though prior studies have focused on single-leg STS, biomechanical insights from those works (e.g., DOI: 10.1519/JSC.0000000000004489; DOI:

10.1080/1091367X.2024.2377096) may help frame STS as a multi-joint, power-demanding, neuromuscular task rather than merely a repetition count.

Response: Thank you for this suggestion. We prefer not to describe the biomechanical and physiological characteristics of the sit-to-stand movement in detail, as the INTRODUCTION section, incorporating all other suggestions, is already 2.5 pages long. We also expect that interested readers are mostly clinical researchers and healthcare professionals working with individuals with PCC, not necessarily (exercise) physiologists, movement scientists or experts in biomechanics. Nevertheless, we have clarified that the sit-to-stand movement is a complex neuromuscular task and depends on several factors, including but not limited to lower limb muscle function.

The new paragraph in the INTRODUCTION reads (pages 3-4): "*The measurement of functional capacity is recommended for people with PCC and part of a core outcome set [11]. Functional capacity is typically assessed as part of (individualised) exercise-based rehabilitation programmes, which demonstrate clinically relevant improvements in cardiopulmonary function and exercise capacity [12,13]. Several tests are available for assessment of functional exercise capacity in people with PCC, of which sit-to-stand tests have received growing attention among healthcare professionals [6,14–17]. In general, sit-to-stand tests assess an individual's ability to perform as many sit-to-stand repetitions as possible within a given time. These tests provide insights into lower limb muscle function; however, test performance also depends on several other factors including balance, coordination, neuromuscular control, pacing strategies, symptom expectation, and motivation. Although the test may appear simple, it is a complex multi-joint neuromuscular task".*

At several points, the text implies that 30s-STS reflects muscle weakness. However, STS performance is influenced by multiple factors including balance, coordination, pacing strategy, symptom anticipation, motivation, and psychological factors. It would strengthen conceptual clarity to explicitly define the construct the 30s-STS is intended to represent in PCC (e.g., lower-limb functional endurance, integrated functional capacity).

Response: This is a great comment. Please see our response to your previous question. We have added another paragraph to the METHODS section (2.4.2 Construct validity) to strengthen conceptual clarity about the test in people with PCC.

It reads (page12): "In addition to PCC-related symptoms and habitual physical activity, performance on functional tests is influenced by a range of factors, including lower limb strength, neuromuscular control, flexibility, pain, motivation, cardiopulmonary fitness, and autonomic function.

As closely related objective measures of lower-limb muscle function were not available, convergent validity could only be assessed indirectly. Therefore, the examined variables were expected to show weak correlations, reflecting related but distinct constructs".

You mention that the 30s-STS is less rigorously validated than the 1-min STS in chronic respiratory disease (p2, l69–70). Please add a short rationale for selecting the 30s protocol (e.g., symptom tolerance, PEM concerns, clinical feasibility).

Response: Thanks for this excellent comment. The decision was informed by people with lived experience. We added the following sentences to the INTRODUCTION section. It reads: *“Our study was developed with input from individuals with lived experience, who raised concerns about study participation when individuals with PEMS are exposed to physical stress tests. Initially, we had planned to use the 1-min STS to assess functional capacity. However, rather than omitting functional testing altogether, we agreed on an alternative and selected the shorter 30-second version”*.

Clarify why MID estimation is particularly critical in PCC research and why COPD-derived MIDs cannot be assumed transferable without validation.

Response: Thank you for this valuable input. We added the following paragraph to the INTRODUCTION section: It reads: *“Recently, a minimal important difference (MID) of two repetitions has been proposed for the 30-s STS test in people with chronic obstructive pulmonary disease [19], a patient population that shares certain clinical features with individuals with PCC, including fatigue, impaired exercise capacity, and lower limb muscle weakness. However, the two populations differ in disease course, symptom variability, clinical predictability, and treatment options. In addition, chronic obstructive pulmonary disease predominantly affects older individuals, whereas PCC also affects younger and middle-aged adults, and a higher proportion of females are typically affected by PCC compared with chronic obstructive pulmonary disease. These demographic and clinical differences may limit the direct transferability of the proposed MID to individuals with PCC.”*

Methods

The Methods section is detailed and generally well organized. However, several important refinements are required.

Since this is a supplement trial, expected changes may be small. This directly impacts anchor-based responsiveness analysis. Please clarify early in Methods that the parent intervention was not primarily designed to improve functional capacity, which may constrain responsiveness and anchor validity.

Response: We added the following part to the METHODS section (2.1, page 5).

It reads: *“Data were collected as part of the PYCNOVID trial [20]. This randomised controlled trial investigated the effects of a nutritional supplement with antioxidative and anti-inflammatory properties on health status (primary endpoint) in people with PCC. Notably, this parent study was not designed to improve functional capacity; it was a secondary endpoint in the main trial, which may have limited responsiveness and anchor validity.“*

PCC diagnosis requires clearer operational definition. While “physician’s diagnosis” is mentioned (p4, l95–96), alignment with WHO criteria (symptom duration, exclusion of alternative diagnoses) should be explicitly stated for international readership.

Response: We fully agree with the reviewer. We extended the paragraph to make this clear, see METHODS (section 2.2, pages 5-6).

It reads: "Individuals with PCC experiencing at least one persistent PCC symptom, such as fatigue, cognitive impairment ("brain fog"), dyspnoea, or PEM [20] were eligible to participate if they were aged 18 years or older, fluent in German, had no planned changes in medication, and had a confirmed SARS-CoV-2 infection verified by polymerase chain reaction (PCR), a rapid antigen test, or a physician's diagnosis of PCC. We applied the World Health Organisation definition of PCC [21] and assessed each participant's clinical history, including the timing of SARS-CoV-2 infection(s), vaccination(s), and the onset and progression of symptoms over time. Specifically, participants were required to have persistent symptoms for ≥12 weeks after SARS-CoV-2 infection that could not be explained by an alternative diagnosis. In the absence of a diagnostic biomarker, each case was carefully adjudicated based on medical report. Exclusion criteria comprised severe comorbidities such as renal failure or advanced heart failure, acute infections, untreated or unstable psychiatric disorders, recent COVID-19 vaccination (<4 weeks prior to baseline or during the study), intolerance to or regular use of the investigational product Pycnogenol®, or participation in another intervention study."

PEM is assessed via a Likert question (p4, l101–103). For Q1-level rigor, justify this choice within Methods and acknowledge measurement limitations earlier rather than only in Discussion.

Response: We added the following sentence to the methods section (2.3.1): It reads: *"We did not assess PEM using a validated instrument, which limits the validity of our evaluation of this complex symptom".*

Screening is described as a "familiarisation test" (p4, l113–114), yet conclusions later state that familiarisation is not necessary. Consider clarifying that screening served as an initial trial, and analyses were conducted to determine whether a separate familiarisation session was required.

Response: Thank you. The revised sentence reads (page 7): *"The 30s-STS test was conducted three times during the study: at screening (initial trial to familiarise participants with the test procedures), at baseline and at the final visit 12 weeks post baseline."*

We further clarified in the analysis section that we aimed to determine whether a familiarisation test is necessary. It reads (page 9): *"To assess test-retest reliability, we compared the 30s-STS results between screening and baseline using Bland-Altman plots and calculated one-way intraclass correlation coefficients (ICC) for consistency. As the screening served as an initial test trial, this analysis also allowed us to examine whether a familiarisation test is necessary".*

Section 2.3 contains extensive information spanning multiple constructs. Consider subdividing into clearer subsections (e.g., Symptom Measures, Functional Testing, Physical Activity Assessment) to improve readability and conceptual clarity.

Response: Done. Thank you.

The definition of submaximal effort (“fear of symptom exacerbation,” p5, l123–125) is clinically important. Please clarify how this variable was coded (binary, ordinal?) and whether standardized instructions were used to reduce reporting bias.

Response: After each test, we asked participants if they gave maximal effort or not (“yes”, “no”). The variable “fear of symptom exacerbation” was coded as binary. The study team underwent rigorous training, and working instructions including standardised wording was in place to minimise bias.

Page 8: *“After each test, we asked whether the participant had exerted maximal effort or had limited their performance out of concern that overexertion might exacerbate their symptoms (herein referred to as submaximal test). Responses were recorded as yes or no. Team members supervising the 30s-STS test underwent rigorous training prior to the start of the study. Standardised working instructions were provided to ensure consistent test administration and minimise bias. We aimed to have the same outcome assessor supervise each participant throughout the 12-week study; however, this was not always feasible”.*

The statistical section is comprehensive and well structured. However, the ICC model choice requires stronger justification. You report a one-way ICC for consistency (p6, l151–152). For test–retest reliability under identical raters and protocol, many readers expect ICC(2,1) or ICC(3,1) with explicit justification (two-way random vs mixed; absolute agreement vs consistency). Please clarify model assumptions and whether systematic bias between trials was considered clinically relevant.

Response: Thank you for the kind words about the statistical methods section. The 30s STS measurements were supervised by different members of our team with standard operating procedures in place, minimising bias. We attempted to ensure that the same outcome assessor supervised each participant throughout the 12-week study; however, this was not always possible.

The specific thoughts behind the selection of ICC(1,1) were as follows: ICC(3,1) is only suitable if agreement between specific raters is desired, and this was not the case here. Our measurement of choice would ideally be ICC(2,1), because it allows for the generalization to the whole population. Unfortunately, it assumes that the measurements of each participant are rated by the same set of raters and that was not the case in our study. For this reason, we opted for ICC(1,1). In this case, the assumption is that the (different) raters are a random sample of the general population of possible raters.

To be on the safe side, we additionally calculated ICC(2,1) for comparative purposes. It showed a value that was quite similar to ICC(1,1): ICC(1,1) of 0.817, ICC(2,1) of 0.824, both rounded to 0.82.

We added a sentence to the METHODS section explaining our rationale for choosing ICC(1,1):

Page 11: *"We opted for the one-way ICC instead of the two-way ICC because the latter assumes that all participants' measurements were supervised by the same outcomes assessors, which was not the case in our study."*

Results

The results and tables are generally well presented and clear.

The use of <5th percentile as an impairment threshold is reasonable, but please justify the clinical reasoning for selecting the 5th and 2.5th percentiles.

Response: The 5th and 2.5th percentiles are standard impairment thresholds in clinical research. The 5th percentile ($z = -1.645$), for example, is widely used as the lower limit of normal for lung function, while the 2.5th percentile is often used for screening in population studies. The choice of percentile depends on the pre-test probabilities. Since our study targets a clinical population with symptoms, we decided to show the 5th percentile only.

Page 9: *"Z-scores for the 30s-STS repetitions were calculated from the data of the sarcopenia cohort from Copenhagen [33]. We use the 5th percentile (z-score -1.645) as a commonly accepted impairment threshold for descriptive purposes only; it is not intended as a diagnostic cut-off"*.

The submaximal effort group showed lower z-scores (p11, l246–249), which is expected. However, stating that there were "no relevant differences" between groups (p11, l250–252) raises an interesting conceptual issue. Fear or pacing behavior itself may represent a meaningful PCC phenotype and deserves deeper interpretation.

Response: The reviewer raises an important point. In the revised manuscript, we discuss the clinical implications (validity) for the group of individuals with submaximal performance due to fear of symptom exacerbation. Although fear or pacing behaviour during the 30s-STS could represent a distinct PCC phenotype, our study was not designed to formally identify or classify such subgroups. We have addressed the implications of submaximal effort on test validity and potential underestimation of functional capacity; further investigation of phenotypic variation would require dedicated studies. We highlight this potential PCC phenotype in our manuscript and call for future research to explore and characterise this subgroup.

The new paragraph reads (pages 20-21): "*Importantly, almost one quarter reported limiting their effort out of fear that symptoms might exacerbate, influencing their 30s-STS results. This group had remarkably lower 30s-STS z-scores compared to those who reported maximum effort (-2.11 versus -1.23). We did not identify any characteristics that explain these findings, such as more severe symptom burden, lower physical activity levels, or presence of chronic disease(s) other than PCC. The two groups were*

well comparable with respect to demographic and clinical characteristics and had reported similar quality of life (Table S2). Notably, this aspect is important to consider because some people with PCC may be capable of performing better in the 30-s STS but chose not to due to fear of symptom exacerbation ("crashes"). Consequently, submaximal effort may lead to an underestimation of individual functional performance, with implications for the validity of the test results at the individual level and their clinical interpretation. From a clinical perspective, it is therefore important to assess fear of symptom exacerbation as part of exercise testing in people with PCC. While this group may present a distinct clinical phenotype, our data do not allow formal identification and detailed characterisation such subgroups. Further research is needed to explore these potential phenotypes and their impact on functional assessment and clinical outcomes".

Please reconcile the "good ICC" with the relatively wide limits of agreement. ICC can be inflated by large between-subject variability. Reporting SEM, MDC95, or coefficient of variation would strengthen clinical interpretability.

Response: Thank you for this comment. It is true that the limits of agreement are comparably wide. We additionally calculated the SEM and MDC95: SEM = 2.35, MDC95=4.25. We added this information to the paper and discuss its being in line with the limits of agreement:

Page 11: *"Additionally, we calculated the standard error of measurement (SEM), defined as the standard deviation at screening times the square root of 1 minus ICC, and the minimal detectable change (MDC95), defined as 1.96 times the square root of 2 times SEM. The calculation of these two values provides additional information about the measurement error and its influence on change."*

Page 16: "The SEM was 2.35 repetitions, with a corresponding MDC95 of 4.25 repetitions, indicating that changes greater than four repetitions can be interpreted as exceeding measurement error.".

Page 20: "However, measurement error and between-subject variability can influence the results and should be considered when interpreting 30s-STS results.

We further added a statement regarding potential learning. Based on the proposed analysis (SEM) and given the large interindividual variability, we decided to be more cautious in that regard.

Page 20: "However, given the large interindividual-variability (wide limits of agreement and a measurement error of ±2.35 repetitions) in a clinical population with fluctuation symptoms, a familiarisation test seems clinically meaningful.

Discussion

The Discussion is generally well structured and demonstrates thoughtful interpretation. The absence of a learning

effect is well addressed, and caution regarding single-item PEM measurement is appropriate.

However, several refinements would strengthen scientific depth:

You state that symptom severity may not limit short functional tasks (p18, l51–53). This requires more nuanced interpretation. Consider discussing:

(i) Pacing and anticipatory behavior decoupling symptoms from performance

(ii) The possibility that 30s duration may not trigger PEM in some individuals

(iii) Temporal mismatch between symptom measurement windows and test performance

Response: Thank you very much for providing such in-depth discussion points. We added the points, as suggested.

It reads (pages 21-22): "Our participants reported high burden of PCC-related symptoms, in particular fatigue (82.7%) and PEM (94 %). Relationships between the 30s-STS test and frequent PCC-related symptoms were weak, suggesting that symptom severity is not necessarily a limiting factor for a short and specific functional task such as the 30s-STS test. Several factors may contribute to this decoupling. First, participants may adopt pacing or anticipatory behaviours, consciously or subconsciously limiting effort to avoid symptom exacerbation, which can mask the expected relationship between symptoms and performance. Second, although some individuals may fear symptom worsening, it is unclear whether such a brief, 30-second task is sufficient to trigger PEM. Finally, there may be a temporal mismatch between the symptom measurement windows and the acute performance during the test, such that momentary functional capacity is not fully captured by symptom questionnaires.

The results of previous studies investigating relationships between 30s-STS performance and PCC-related symptoms are heterogeneous [6,16,17]. Our data are consistent with a previous study of 102 individuals with PCC showing no relationships between the 30s-STS test and fatigue, PEM, myalgia and dyspnoea [16]. We studied a heterogenous group of people with PCC, and between-subject variability in those reporting fatigue (herein defined as FACIT-Fatigue scores <34) was wide, ranging from -4 to 2 z-score units. With respect to PEM, as expected, the group of participants reporting very severe PEM had lower 30s-STS z-scores compared to those reporting either mild, moderate or severe PEM. However, these findings must be interpreted with caution since we did not use a validated instrument to assess PEM [22] but rather a single question with a Likert-type severity grading, which has limited validity. Taken together, these considerations suggest that short-duration functional tests provide complementary information to symptom assessments but may not fully reflect the impact of PCC-related symptoms on daily life".

Justification of Danish reference values based on similar inactivity prevalence (p20, l103–112) is reasonable but indirect. Consider framing this as approximate benchmarking rather than definitive impairment classification. Present raw repetition data as primary, with z-scores as contextual support.

Response: Thank you for the suggestion. We added this point, as suggested.

The new paragraph reads (page 24): *“Limitations include lack of reference values for the 30s-STS from a Swiss population. We therefore used data from a Danish Cohort (Copenhagen Sarcopenia Cohort) [33], a cohort of persons aged 20-93 years to calculate z-scores for test interpretation. We do not believe that the reference dataset introduced systematic error (e.g., under- or overestimation of 30s-STS performance at the population level). Both Switzerland and Denmark are high-income European countries, and the proportion of physical inactivity (i.e., not meeting the World Health Organisation physical activity guidelines) appears to be comparable: Denmark: 28.5% (95% CI, 22.7-35.0) and Switzerland: 29.3% (95% CI, 21.2-38.8) [49]. Given that physical activity and exercise capacity are typically correlated [50], similar levels of physical inactivity support the assumption that these countries have broadly comparable functional capacity profiles. Importantly, use of the Danish reference data and corresponding equations should be considered an approximate benchmark rather than a definitive impairment classification of impairment.”*

Since anchor-based MID was not feasible, strongly consider emphasizing MDC95 as the measurement error threshold, with MID described as provisional.

Response: The reviewer is right in stressing that a relatively large measurement error has an influence on the interpretation of our proposed MID value. We now discuss this and emphasize that our preliminary estimate requires prospective evaluation. We integrated results from MDC95 in the abstract with clear distinction between distribution based MID (provisional) and MDC95 suggested as measurement error threshold.

Page 24: “Distribution-based approaches suggest an MID of approximately two repetitions for the 30s-STS test; however, this preliminary estimate requires prospective evaluation.

Page 25: “Importantly, as the MDC95 of 4.25 repetitions suggests that changes below this threshold might be primarily or partly due to measurement error and between-subject variability, the proposed MID value of two repetitions must be interpreted with caution. A change of two repetitions could fall within measurement error, while changes above 4 repetitions can be interpreted as both real and meaningful. However, using a comparatively small MID of two repetitions for sample size calculations represents a conservative approach in the absence of further evidence: a smaller MID results in a larger sample size, thereby ensuring sufficient power for future studies.

Page 27: “Distribution-based methods suggest a provisional minimal important difference of at least two repetitions, whereas the minimal detectable change is about four repetitions”

Conclusions

The conclusion is concise and appropriately cautious regarding the need for future anchor-based MID validation (p21, l122–124).

Minor correction:

“suggest as minimal” → “suggest a minimal” (p21, l121–122).

Response: Done

Part 1: General reporting recommendations for all studies on measurement properties

Item	Item name	Item description	Comment
Report section: Title<br>T1	Title	Identify the report as a study of one or more measurement properties of a specific PROM to measure a specified construct in a specified population	Measurement properties in title of manuscript
Report section: Abstract<br>A1	Objectives	Provide the specific objective(s) of the research, specifying (1) the name (and version, if relevant), and construct(s) of the PROM, (2) the measurement properties being evaluated, and (3) relevant study characteristics	Abstract provides information that 1) 30s STS is outcome; 2) feasibility, safety, test-retest reliability, construct validity and responsiveness to change were the properties of interest; 3) this is a secondary analysis using data from an RCT
A2	Design	Specify (details of the) study design used to evaluate the measure-ment properties	Secondary analysis using data from an RCT
A3	Methods	Specify the methods for evaluating each measurement property	Bland-Altman plots, ICC, MDC95, correlation coefficients, MID (anchor- and distribution based)
A4	Results	Provide the main results for all measurement properties evaluated	ICC, mean bias MDC95, MID
A5	Discussion/Conclusions	Provide a brief statement of the implications of the findings in the context of existing evidence on the PROM	Good test-retest reliability, suggested MID smaller than MDC95
Report section: Introduction I1	PROM	Specify the name and, if relevant, the version, and construct(s) of the PROM	See introduction
I2	Target population & context of use	Specify the target population and context of use that the PROM was designed for	People with PCC; see introduction
I3	State of knowledge & Rationale	Provide a description of the current scientific knowledge (what is known and not known) regarding the measurement properties of the PROM. Explain why the new study is necessary. Provide citations for the original development paper(s)	See introduction
I4	Objectives	Provide the specific objective(s) of the research, specifying (1) the name (and version, if relevant) of the PROM, (2) the measurement properties being evaluated, and (3) relevant study sample characteristics	See last paragraph of introduction
Report section: General Methods<br>GM1	Study design	Specify (details of the) study design used to evaluate the measure-ment properties	See section «Study design and setting»
GM2	Participants	Specify how the study participants were selected. Specify the inclusion and exclusion criteria	See section “Participants”

GM3	PROM details	Provide details about the original version of the PROM as well as of the PROM version being studied, specify the conceptual framework (reflective/formative model), details on the structure (the number of items and subscales), the language, response scale, recall period, direction of scoring, and scoring algorithm of the PROM. Specify how the PROM was administered (e.g., in what setting, mode of administration (e.g. paper, electronic) what instructions were given), including the country in which it is administered	See section «30-second sit-to-stand test» for information on our outcome measure (not a PROM)
GM4	Additional data collection	Describe why and how other data was collected (e.g., construct and measurement properties of the comparator instruments, characteristics of groups being compared, and rationale for choosing groups), including mode of administration (e.g., paper, electronic)	See sections «Symptoms and patient-reported outcome measures» and «Physical activity»
GM5	Time points procedures	Provide all time points of all measurements	See section «30-second sit-to-stand test»
GM6	Justification for sample size	Provide a rationale for the sample size for all measurement proper-ties analyses (including subgroups)	Secondary analysis of trial data; see section «Study design and setting»
GM7	Statistical analyses	Describe the statistical analyses corresponding to all objectives (see measurement properties specific boxes). Describe the criteria for good measurement properties. Name the statistical package used and the version	See «Statistical analysis» section
GM8	Missing data	Describe approaches for dealing with missing data	See «Statistical analysis» section
GM9	Unplanned analysis	Specify analyses that were unplanned and their rationale	Secondary analysis of trial data; all analysis as described in "Statistical analysis" section
Report section: General results<br>GR1	Participant characteristics	Provide study participants' characteristics, specified per subgroup if applicable	See beginning of results section and Table 1
GR2	Sample size	Provide the total number of participants included in the study and the sample size for each analysis	See results section
GR3	Missing data	Provide amount of (proportion or count) and reasons for missing data for each analysis for the PROM, and for any analyses of other outcome measurement instruments	See beginning of results section, Table 1 and «Feasibility» section
GR4	Results	Describe the results corresponding to all objectives (see measure-ment properties specific boxes)	See results section
Report section: Discussion/conclusions<br>DC1	Measurement property evidence	Provide the main findings and if each measurement property is sufficient or insufficient and why	See discussion
DC2	Practical relevance	Discuss the practical relevance of the findings in terms of recom-mendations for (not) using the PROM	See discussion

DC3	Strengths and limitations	Discuss strengths and limitations of each study. For example, discuss if there were any potential biases in the study that could have impacted the results	See «Strengths and limitations» section
DC4	Generalizability	Discuss generalizability of the results. For example, discuss whether the results could be generalized to other populations given the sample studied	See «Strengths and limitations» section
DC5	Instrument changes	Discuss what modifications are needed to the existing PROM	Not applicable because no PROM, but objective measurement
DC6	Future research	Describe new research questions or hypotheses generated from these findings, and provide/describe the research needed to answer those questions	See discussion and conclusions
DC7	Conclusions	Provide the overall conclusions for the use of the PROM	See conclusions
Report section: Other information<br>O1	Conflict of interest	State any conflict of interest you may have related to the PROM. This may include any involvement in the development of the PROM or any commercial funding or profit	No conflicts of interest.<br>See statement requested from journal.

Part 2: Specific reporting recommendations

Specific Reporting recommendations for studies on Content Validity: Not applicable
Specific Reporting recommendations for studies on Structural Validity: Not applicable
Specific Reporting recommendations for studies on Internal Consistency: Not applicable
Specific Reporting recommendations for studies on Cross-Cultural Validity/Measurement Invariance: Not applicable

Specific Reporting recommendations for studies on Reliability:

Item		Item description	
Reliability: Methods<br>R1	Stability of patients	Provide arguments for assuming stability of patients on the construct of interest between the repeated measurements	See data sources and measurements section
R2	Statistical analyses	Provide the specific intraclass correlation coefficient (ICC) or kappa formula used and justify the choice of the formula used	See statistical methods section
R3	Sources of variation	Specify which source of variation was purposely varied across the repeated measure- ments	No source of variation varied on purpose; secondary analysis of trial data
R4	Time interval	Provide arguments for the appropriateness of the time interval	See data sources and measurements section
Reliability: Results<br>R5	Stability of patients	Describe whether patients were stable on the construct of interest between the repeated measurements	See results of test-retest reliability and explanations regarding the time interval between the two measurements
R6	Statistical analyses	Provide results for calculated statistics (ICC or kappa) and measures of precision (standard errors or confidence intervals). Provide results of all variance components, or contingency tables	See results section

Specific Reporting recommendations for studies on Measurement Error:

Item		Item description	
Measurement Error: Methods ME1	Stability of patients	Provide arguments for assuming stability of patients on the construct of interest between the repeated measurements	See methods for test-retest reliability, results section and discussion
ME2	Statistical analyses	Provide and justify the specific statistical formula used for standard error of measurement (SEM), smallest detectable change (SDC), limits of agreement (LoA), or percentage (specific) agreement	See methods for test-retest reliability
ME3	Sources of variation	Specify which source of variation was purposely varied across the repeated measure- ments	No source of variation varied on purpose; secondary analysis of trial data
ME4	Time interval	Provide arguments on the appropriateness of the time interval	See data sources and measurements section
ME5	Stability of patients	Describe whether patients were stable on the construct of interest between the repeated measurements	See results of test-retest reliability and explanations regarding the time interval between the two measurements
ME6	Statistical analyses	Provide results for calculated statistics and measures of precision (if applicable). Provide results of all variance components included in the SEM, provide the systematic change or difference between the repeated measurements (when LoA was applied), or contingency tables	See results section

Specific Reporting recommendations for studies on Criterion Validity: Not applicable

Specific Reporting recommendations for studies on Hypothesis Testing for Construct Validity:

Item		Item description	
Hypothesis Testing for Construct Validity: Methods ConV1	Hypotheses	State hypotheses, and provide the rationale for each hypothesis	See methods section
ConV2	Statistical analyses	Specify all statistical methods used to test the hypotheses	See methods section
ConV3	Results: Statistical analyses	Provide all results and specify if each result is in accordance with its hypothesis	See results section

Specific Reporting recommendations for studies on Responsiveness:

Item		Item description	
Responsiveness: Methods<br>Resp1	Hypotheses	State hypotheses, and provide the rationale for each hypothesis	See sections on construct validity and MID
Resp2	Intervention/Exposure	Provide the intervention given or exposure in the interim period (or report that no intervention was provided)	Secondary analysis of trial data; see methods section
Resp3	Statistical analyses	Specify all statistical methods used to test the hypotheses	See statistical methods section
Resp4	Statistical analyses	Provide results for calculated statistics, and specify, for the construct approach, if each result is in accordance with its hypothesis	See results section
